# Effect of incorporating chitosan/hydroxyapatite composite nanoparticles into a universal adhesive on bonding efficacy to caries-affected dentin and bioactivity

**DOI:** 10.1186/s12903-026-07909-3

**Published:** 2026-03-12

**Authors:** Rana Mamdouh El-Demellawy, Ahmed El-Banna, Mohamed M. Kandil, Dalia I. El-Korashy

**Affiliations:** 1 Biomaterials Department, Faculty of Dentistry, Ain-Shams University, Organization of African unity, Cairo, Egypt; 2 Biomaterials Department, Faculty of Dentistry, Galala University, Suez, Egypt

**Keywords:** Chitosan, Nano-hydroxyapatite, Universal adhesive, Micro-tensile bond strength, In vitro bioactivity, Wettability, PH, Degree of conversion

## Abstract

**Background:**

To evaluate the impact of incorporating chitosan/hydroxyapatite composite nanoparticles (CT/HAP NPs) into a universal adhesive on its bonding effectiveness to caries-affected dentin (CAD) and its in vitro bioactivity.

**Methods:**

CT/HAP NPs (50:50 by weight) were synthesized via a one-step co-precipitation method. All-Bond universal adhesive was modified with either 0.5 wt% or 1 wt% CT/HAP NPs, yielding three groups: unmodified control, 0.5 wt%, and 1 wt% CT/HAP. Forty-eight molar teeth (4 teeth per group) were used to prepare 240 bonded beams using either etch-and-rinse (ER) or selective-etch (SE) mode (*n* = 20 bonded beams per group). Micro-tensile bond strength (µTBS) test was conducted using a universal testing machine after 24 h and 6 months of storage in simulated body fluid (SBF). Fifteen adhesive discs (5 mm x 1 mm) (*n* = 5) were examined using an environmental scanning electron microscope combined with an energy-dispersive X-ray spectroscopy **(**ESEM/EDX**)** following storage in SBF for 1 day and 28 days to assess bioactivity and Ca/P ratio. Wettability, pH, and degree of conversion (DC) were assessed using a digital light microscope, a pH meter, and Fourier transform infrared (FTIR) spectroscopy, respectively. µTBS and wettability data were investigated utilizing multilevel ANOVA, while pH and DC were evaluated utilizing one-way ANOVA; Tukey’s post hoc test was applied for pairwise multiple comparisons. The significance level was set at (*p* < 0.05).

**Results:**

The 1 wt% CT/HAP group exhibited significantly higher µTBS at 24 h (ER: 37.50 ± 6.90 MPa; SE: 32.52 ± 6.61 MPa) and at 6 months (ER: 41.81 ± 7.64 MPa; SE: 42.10 ± 7.23 MPa) than the 0.5 wt% CT/HAP and control groups. Adhesives containing CT/HAP NPs showed improved surface mineral deposition and higher Ca/P ratios compared to control. Wettability, pH, and degree of conversion revealed no significant changes.

**Conclusions:**

Incorporating chitosan/hydroxyapatite composite nanoparticles into an ultra-mild universal adhesive enhanced its bonding efficacy to caries-affected dentin as well as its bioactive potential, without jeopardizing the physical and chemical properties of the adhesive.

## Background

The most frequent pathological condition affecting the integrity of tooth structure is dental caries. Two layers of different histological and chemical structures form the carious dentin [1]. The outermost layer is the caries-infected dentin (CID), which is bacterially infected and non-remineralizable. In contrast, the innermost layer, the caries-affected dentin (CAD), is uninfected and can be remineralized [1, 2]. 

Minimally invasive dentistry (MID) involves the selective removal of the CID while preserving the CAD [3]. However, there are challenges to bonding to CAD, including diminished mechanical properties, reduced mineral content, and excess water, all of which negatively impact the strength and longevity of resin-dentin bonding, regardless of the adhesive system applied [4]. Different strategies have been employed to overcome these challenges, including collagen crosslinking, MMP inhibition, and biomimetic remineralization [1, 5, 6]. 

Chitosan (CT) is a natural polysaccharide derived from the N-deacetylation of chitin. It has attracted substantial attention in the biomaterials field because of its biocompatibility, antibacterial properties, and the ability to chelate calcium ions (Ca²⁺) through its amino and hydroxyl functional groups [7]. Moreover, chitosan and its derivatives (e.g., phosphorylated chitosan and carboxymethyl chitosan) have demonstrated the ability to mimic the bio-remineralization of collagen fibers in dentin [8, 9] and to establish cross‑links with type I collagen, thereby enhancing resin–dentin bond strength and hybrid layer stability, particularly in CAD [10]. 

Hydroxyapatite (HAP) is the main mineral phase of dentin, providing its rigidity and inorganic structure [11]. However, when reduced to the nanoscale, nano-hydroxyapatite (nHAP) exhibits greater surface area and higher solubility [12], which are advantageous for bioactivity and remineralization by enhancing ion release and promoting apatite nucleation and mineral deposition [11]. 

Accordingly, Chitosan/hydroxyapatite (CT/HAP) composite systems have emerged as promising biomimetic materials for dentin remineralization [13], combining the collagen-interactive properties of chitosan with the bioactive and mineralizing potential of nHAP [14]. 

In the context of dental adhesive systems, previous studies have reported that dental adhesives modified with chitosan alone can enhance bonding efficacy and improve hybrid layer resistance to enzymatic and hydrolytic degradation [10, 15]. Concurrently, other studies have incorporated nano-hydroxyapatites as fillers in adhesives, demonstrating their potential to encourage the remineralization of demineralized dentin and boost the bioactivity of adhesives, thereby improving the longevity of resin-dentin bonding [16–18]. 

Despite these promising findings, no studies have yet investigated the impact of incorporating chitosan/hydroxyapatite composite nanoparticles into universal adhesive systems, aiming to integrate their complementary effects within a single adhesive formulation to enhance dental bonding performance, particularly in caries-affected dentin.

Therefore, the present study aims to evaluate the effects of incorporating two concentrations of CT/HAP composite nanoparticles into a universal adhesive on its micro-tensile bond strength (µTBS) to caries-affected dentin after 24 h and 6 months of storage in simulated body fluids (SBF) with different etching modes, as well as its in vitro bioactivity, wettability, pH, and degree of conversion (DC). Two null hypotheses were tested: the first null hypothesis was that adding CT/HAP NPs would not affect the bond strength and durability of the universal adhesive to caries-affected dentin when applied with different etching modes, and the second null hypothesis was that adding CT/HAP NPs would not influence the bioactivity of the adhesive.

## Methods

A detailed overview of the materials utilized in the present study, including their description, composition, manufacturer, and lot number, is provided in (Table 1).

**Table 1:** Materials used in the current study, their description, composition, manufacturer, and lot number

Brand name	Description	Composition	Manufacturer	Lot number
All-bond Universal	Ultra-Mild Universal Adhesive System (pH > 3)	10-MDP, Bis-GMA, HEMA, Initiators, Ethanol, Water.	Bisco Inc., Schaumburg, IL, USA	2,200,007,024
Grandio	Universal Nano-hybrid Dental Resin Composite, Shade A2	Bis-GMA, TEG-DMA, UDMA, Bisphenol A, Polyethylene glycol, Di-ether, Di-methacrylate 59% vol., 78.5% by weight, Silica nanofiller (5–75 nm), zirconia /silica nanoclusters (0.6–1.4 μm)	VOCO GmbH, Cuxhaven, Germany	2,250,322
Meta Etchant	Acid-Etchant	37% phosphoric acid semi-gel	Meta Biomed, Korea	MET2211231
CT/HAP NPs	White, opaque needle-shaped NPs	50:50 wt% composition of chitosan and hydroxyapatites	Nanogate, Cairo, Egypt.	

*10-MDP* 10-methacryloyloxydecyl dihydrogen phosphate, *Bis-GMA* Bisphenol A-glycidyl methacrylate, *HEMA* 2-hydroxyethyl methacrylate, *TEGDMA* Triethylene glycol dimethacrylate, *UDMA* Urethane dimethacrylate

### Preparation of CT/HAP composite nanoparticles

CT/HAP NPs with a 50:50 by weight were prepared using the one-step co-precipitation method [14, 19]. One gram of chitosan powder (low-MW: 50,000-190,000 Da with 85% of degree of deacetylation (DD), Sigma-Aldrich, Saint Louis, USA) was dissolved in 100 mL of 1% acetic acid and mechanically stirred at 2500 rpm (HM100-S overhead stirrer, Henan, China) for 24 h to form a transparent solution with a 1% (w/v) concentration. 20 mL of 0.5 M calcium nitrate tetrahydrate (Ca (NO_3_)_2_.4H_2_O, MilliporeSigma, Burlington, MA, USA) was introduced to the chitosan solution under stirring, followed by the dropwise addition (3 mL/min) of 12 mL of 0.5 M di-ammonium hydrogen phosphate ((NH_4_)_2_. HPO_4,_ MilliporeSigma, Burlington, MA, USA) to achieve a Ca/P molar ratio of 1.67, leading to the formation of CT/HAP nanoparticles with a weight ratio 50:50. The mixture was stirred continuously for 24 h and then sonicated in an ultrasonic cleaner (Memory Quick, KWUN WAH International LTD, China) at 20,000 rpm for 3 h to ensure homogeneity and reduce the average particle size [15]. The pH was continuously monitored throughout the entire process with a digital pH meter. After standing overnight to remove air bubbles, the pH was raised to 11 with NaOH, resulting in precipitation. The resulting precipitate was centrifuged at 3000 rpm for 30 min (Z-36HK, Hermle, Germany), washed, and then frozen at − 18 °C. The precipitate was subsequently freeze-dried at − 50 °C for 48 h (FDE-0350, Korea) to yield a white, opaque powder [19]. 

### Characterization of CT/HAP composite nanoparticles

Phase composition and chemical structure of the prepared CT/HAP composite nanoparticles were characterized using X-ray diffraction (XRD) and Fourier transform infrared (FTIR) spectroscopy, respectively. All measurements were performed in triplicate to ensure reproducibility [20]. 

Phase composition and the crystalline structure of CT/HAP, pure CT, and nHAP powder were analyzed via a powder X-ray diffractometer (XRD, D8 Discover, Germany) using Cu Kα radiation at 30 mA and 45 kV over a range between 5° and 90°. Phase identification was performed using the ICDD database (International Centre for Diffraction Data, Pennsylvania, PA, USA) [16]. 

Chemical characterization of each tested powder was analyzed using FTIR spectroscopy operated in Attenuated Total Reflectance (ATR) mode (ALPHA II, Bruker, Germany). The specimens were irradiated with infrared light at wavelengths between 400 and 4000 cm^− 1^, and the absorbance spectra of the samples were recorded [21, 22]. 

### Preparation of the experimental adhesive

CT/HAP NPs were incorporated into a commercial adhesive (All-Bond Universal, Bisco, Schaumburg, USA) to prepare two modified groups containing 0.5% and 1% (w/v) CT/HAP NPs, based on findings from a prior pilot study, to be compared with an unmodified adhesive (control group). For the 0.5 wt% CT/HAP formulation: 0.005 g of CT/HAP NPs was accurately weighed using a 4-digit analytical balance (AZ 214, accuracy ± 0.0001 g; Sartorius AG, Germany) and added to 1 mL of the adhesive in a cleaned, light-proof container [23]. Similarly, 0.010 g of CT/HAP NPs was used for the 1 wt% CT/HAP formulation. Each mixture was subjected to ultrasonic agitation in a bath (MCS, Codyson, China) at room temperature for 20 min to ensure uniform dispersion [23]. The modified adhesives were re-homogenized by ultrasonication before each use to maintain nanoparticle dispersion [15, 23]. 

### Sample size calculation and grouping

All-Bond Universal adhesive was modified with two concentrations of CT/HAP NPs, resulting in three experimental groups: one unmodified, control group (group I), and two modified groups containing 0.5 wt% CT/HAP (group II) and 1 wt%. CT/HAP (group III). Sample size was calculated using R statistical software (version 4.5.0) on Windows, with an alpha (α) level of 0.05, and a beta (β) level of 0.2, corresponding to 80% statistical power.

For the µTBS test, based on a pilot study, the minimal required sample size was 80 bonded beams per group, with a total of 240 specimens. Each group was further subdivided according to the etching mode: etch-and-rinse (ER) or selective-etch (SE) modes, and storage time (24 h or 6 months), resulting in 20 bonded beams for each experimental condition. The tooth/beam clustering was treated as a random effect in the multilevel ANOVA.

For wettability testing, based on previous studies [15, 24], the minimal required sample size was identified to be 10 specimens per group, with a total of 30 specimens. However, the number was increased to 42 specimens (14 per group) to ensure accuracy and to compensate for potential laboratory errors. Each group was further subdivided according to the etching mode (*n* = 7).

For pH and degree of conversion (DC) assessment, based on previous studies [15, 23, 24], the calculated sample size was identified to be 15 specimens for each test (5 per group). However, the number was increased to 21 specimens (*n* = 7) to ensure accuracy and to compensate for potential laboratory errors.

**Fig. 1:**
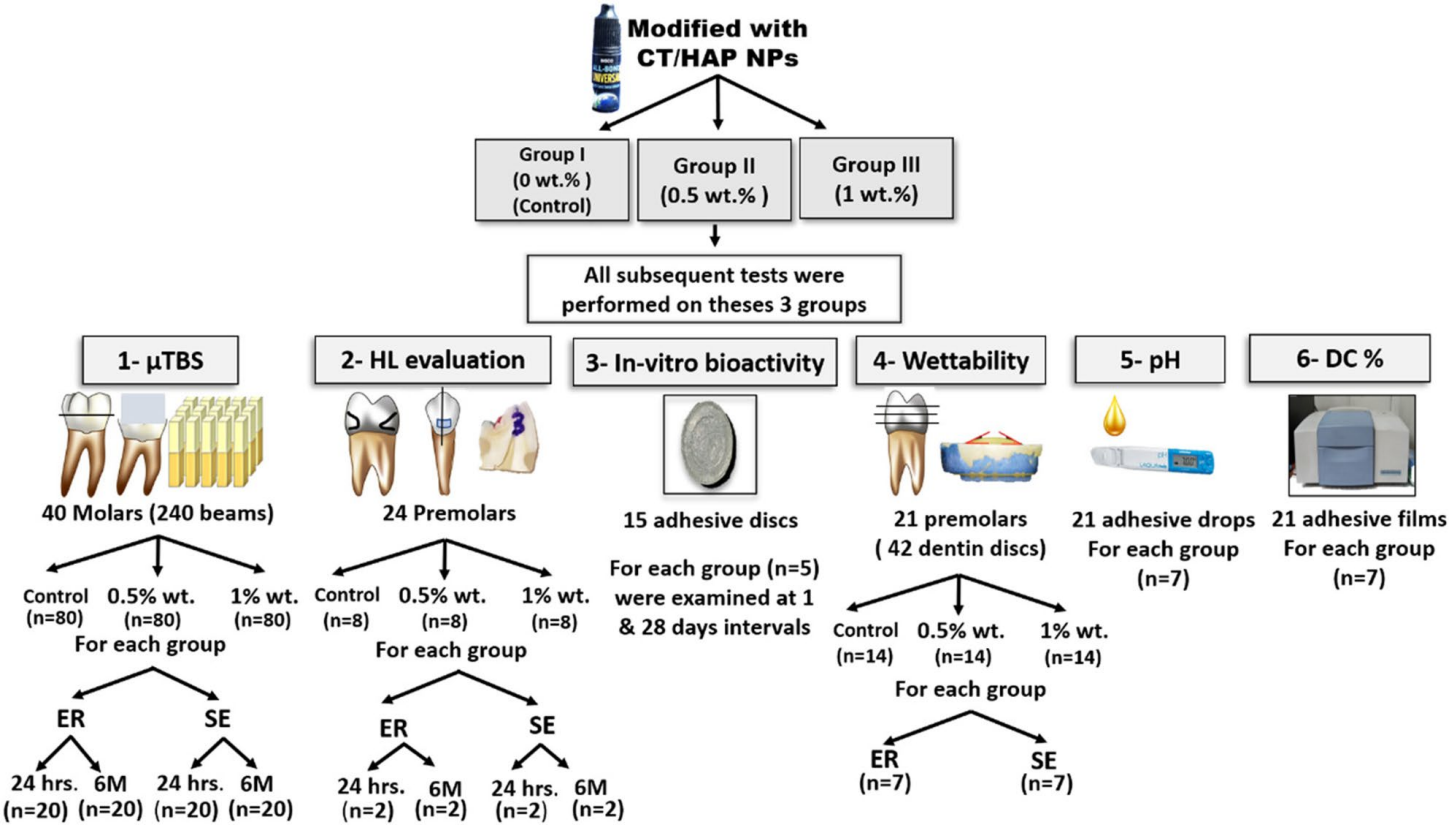
A schematic flowchart of the current study design

For the qualitative evaluation of the hybrid layer (HL), 24 premolars were used (8 per group). Each group was further subdivided according to the etching mode (ER or SE) and storage time (24 h or 6 months), resulting in two premolars for each experimental condition [15]. For the descriptive in vitro bioactivity assessment, 15 adhesive discs were prepared (5 per group) and examined at two time intervals: 1 day and 28 days [23]. A schematic flowchart of the current study design is presented in (Fig. 1).

## µTBS testing and bond durability

### Teeth preparation

Forty-eight sound, non-carious human third permanent molars were collected from patients aged 20 to 40 years old, after extraction for orthodontic reasons at the Faculty of Dentistry, Ain Shams University, following the approval of the ethical committee (FDASU–RecED032128). The teeth were ultrasonically cleaned and inspected under a stereomicroscope at 25x magnification to verify the absence of any cracks, fractures, carious lesions, or any structural defects. Teeth were then stored in distilled water containing 0.5% thymol at 4℃ for not more than one month [15]. Occlusal enamel surface of each tooth was decoronated, using a precision diamond saw (Isomet 4000, Buehler, Lake Bluff, IL, USA) under water coolant, to obtain a flat mid-dentin surface, which was confirmed under the stereomicroscope to ensure complete enamel removal. The saw was operated at a blade speed of 2600 rpm and a feed rate of 8.5 mm/min [23, 25]. 

Molar apices were horizontally sectioned to facilitate the flow of SBF through the root canals and into the dentinal tubules [23, 26]. This was followed by grinding the dentin surfaces with 600-grit silicon carbide paper (SiC) under water coolant for 1 min, then rinsing to replicate the clinical smear layer [25]. The outer margins of the tooth surface were protected with two layers of acid-resistant nail polish, leaving a rectangular, flat dentin window uncovered to ensure that only dentin was exposed to the demineralizing solution. Caries-affected dentin was then induced by immersing each tooth in a demineralizing solution (50 mM acetate buffer, pH 4.5, with 2.2 mM CaCl_2_, 2.2 mM KH_2_PO_4_, 0.5 ppm NaF, and 50 mM lactate gel) prepared at the Faculty of Pharmacy, Ain Shams University, according to Smith et al. [27] Each tooth was immersed for 4 days at 25^°^C without changing the solution [25]. The required volume of demineralizing solution for each tooth was calculated by multiplying the surface area of the exposed dentin window by two [28]. After demineralization, teeth were washed with deionized water for 15 min, followed by ultrasonic cleaning and gentle drying to remove any residues [23, 25]. Teeth were randomly assigned to each experimental group, with four teeth allocated per condition in accordance with the Dental Materials guidelines [29]. 

### Bonding procedure

A metal matrix clamp (No. 1.542 Tor VM, Russia) was placed around each tooth for a 4 mm composite build-up. Bonding protocols were applied according to the manufacturer’s instructions. In etch-and-rinse groups, enamel margins were etched with 37% phosphoric acid for 30 s, and the dentin surface for 15 s, followed by rinsing and blot-drying to maintain dentin moisture. For the selective-etch groups, only enamel margins were etched with 37% phosphoric acid for 30 s, followed by rinsing and blot-drying [23]. 

Two coats of universal adhesive were applied to dentin with a rubbing motion for 20 s each, gently air-dried for 10 s, and light-cured for 10 s using LED light-curing unit (Elipar S10, 3 M ESPE, Germany) at an output intensity of 1200 mW/cm². Resin composite was applied in 2-mm increments; each increment was light-cured for 20 s, and the final layer was covered with a glass slide on a celluloid matrix during curing to ensure a smooth surface and prevent oxygen-inhibited layer formation. Specimens were stored individually in polyethylene tubes containing SBF (pH 7.4), prepared at the Faculty of Pharmacy, Ain Shams University, according to the following formula: [25, 30] (0.231 g K_2_HPO_4_.3H_2_O, 8.035 g NaCl, 0.355 g NaHCO_3_, 0.311 g MgCl_2_.6H_2_O, 0.225 g KCl, 0.072 g Na_2_SO_4_, 39 mL of 1.0 M HCl, 0.292 g CaCl_2_, 0–5 mL of 1.0 M HCl with 6.118 g Tris) in an incubator at 37 °C for either 24 h or 6 months [23, 31]. The storage solution was renewed weekly [23]. 

### µTBS testing

Roots were embedded in chemically cured acrylic resin blocks, and the teeth were sectioned longitudinally in both the X- and Y-axes directions using the Isomet diamond saw to obtain bonded beams. Each beam measured about 1 × 1 mm (± 0.1 mm), and its dimensions were verified with a digital caliper (Series 500, Mitutoyo, IL, USA) with an accuracy of ± 0.001 inch (0.0254 mm). Beams shorter than 4 mm in dentin height were discarded. Five central bonded beams were obtained from each tooth and then fixed to the upper and lower portions of a custom-made metallic attachment using a fast-setting cyanoacrylate adhesive, with the bonded interface oriented perpendicular to the applied load [23]. µTBS was measured by a universal testing machine (Instron 3365, Norwood, MA, USA). Tensile load was applied at a crosshead speed of 1 mm/min until failure. µTBS was computed automatically in (MPa) using the BlueHill3 software by dividing the failure load by the cross-sectional area of the bonded interface (mm²) [15, 32]. 

After debonding, the fractured interface of each specimen was examined using a stereomicroscope (Olympus Stereozoom SZ 40 Microscope, Tokyo, Japan) at 40x magnification to determine the failure mode. Failure modes were classified as adhesive (at the dentin-resin interface), mixed (failure occurred in the adhesive layer and included one of the substrates), cohesive in dentin, or cohesive in resin composite [15]. 

### Hybrid layer (HL) evaluation

Class V cavities were prepared buccally and lingually in 24 caries-free first premolar teeth (*n* = 2). Grouping, dentin demineralization, and bonding procedures were carried out following the same protocol used for µTBS testing. The bonded teeth were maintained in SBF at 37℃ for either 24 h or 6 months. To prepare the teeth for scanning electron microscopy, each bonded tooth was sectioned buccolingually into two halves using the Isomet diamond saw. Each half was smoothed using SiC papers with sequential grits of 400, 600, and 1000 under water, followed by cleaning with 37% phosphoric acid for 5 s and thorough rinsing for 30 s. The halves were placed in 5% NaOCl for 10 min, followed by rinsing for 5 min, and dehydration in 99% ethanol for 5 min. The sectioned interface surface was sputter-coated with gold (Hummer 8, Ladd Research, USA) for 90 s at 15 mA, and examined under scanning electron microscopy (SEM) at magnifications of 1000x and 2000x at an accelerating voltage of 15 kV [15]. 

### In vitro bioactivity assessment

Disc-shaped specimens (5 mm diameter x 1 mm thickness) were fabricated using a split Teflon mould. Adhesives were applied to a celluloid matrix above a glass slide until the mould was filled, then covered with another glass slide on top of the celluloid matrix and light-cured for 40 s using an LED light-curing unit [23, 31]. The adhesive discs were stored in the prepared SBF in an incubator at 37 °C and examined after 1 and 28 days time interval. Examination of each specimen was done without any surface modification or coating, using an environmental scanning electron microscope (ESEM) combined with an energy-dispersive X-ray (EDX) spectroscopic unit (Inspect™, FEI Company, Netherlands) at 1500x magnification to determine elemental analysis and Ca/P molar ratios.

### Wettability measurement

Twenty-one first premolar teeth were decoronated using the Isomet diamond saw under water coolant by removing the occlusal enamel surface, including any enamel islands, to obtain a flat mid‑dentin surface, which was verified under a stereomicroscope [25]. Two dentin discs (1.5 mm thick) were produced from each tooth above the CEJ. The surfaces selected for contact angle measurement were standardized by using the lower surface of the first disc and the upper surface of the second disc [15]. 

As described in the µTBS section, a standardized smear layer was created, the outer margins were protected with nail polish, and the teeth were immersed in a demineralizing solution. Afterwards, the teeth were rinsed with deionized water, ultrasonically cleaned, and gently air-dried to remove any residues of solution or polish [23, 25]. 

In the ER groups, dentin surfaces were etched with 37% phosphoric acid for 15 s, rinsed for 30 s, and gently dried to maintain dentin moist, while the SE groups received no prior etching [23]. 

For contact angle measurement, a micropipette was used to apply a drop (3µL) of adhesive on the dentin disc, which was placed on the stage of a digital microscope (Dino-Lite digital microscope, Taiwan) [15]. After 30 s under controlled ambient conditions (25 °C, yellow-filtered light), the contact angle was calculated with Dino Capture 2.0 software (version 1.5.43, Taiwan) by outlining the droplet baseline and tangent. The angles from both sides of each droplet were recorded, and their mean value was taken as the contact angle (θ) of the specimen [15]. 

### pH measurement

The pH of the adhesives was assessed using a pH meter (Horiba LAQUAtwin Compact, OneTemp, Japan). Before measuring, the pH meter was cleaned with distilled water, calibrated with pH 4 and 7 buffer solutions, and any excess water was wiped off. A drop (20 µL) of the adhesive was placed on the meter tip using a micropipette under controlled conditions (25 °C, yellow-filtered light), and the pH reading was recorded within 2 s of sample application [15]. 

### Degree of conversion (DC) measurement

Potassium bromide (KBr) discs were fabricated for each adhesive sample for FTIR analysis. The absorbance spectrum of ambient air was first recorded as a baseline [23]. The control and modified adhesives were applied directly onto the KBr discs via a micro-brush, and the spectra of the unpolymerized adhesives were captured in the mid-infrared (MIR) region. The discs were then photo-cured for 10 s using the LED light-curing unit through a celluloid matrix, and post-curing absorbance spectra were recorded [23]. The degree of conversion (DC, %) was estimated following the baseline method, before and after curing, as follows [33]:

$$\mathrm{DC}\%\;=\;1\;-\;[(\mathrm{R}\;\mathrm{cured}\;/\;\mathrm{R}\;\mathrm{uncured})]\;\times\;100$$, where:

R cured (Aliphatic absorption peak at 1638 cm^− 1^/ Aromatic absorption peak at 1608 cm^− 1^) polymer.

R uncured (Aliphatic absorption peak / Aromatic absorption peak) monomer.

### Statistical analysis

Numerical data were reported as mean ± standard deviation (SD). The Shapiro-Wilk and Levene’s tests were applied to assess normality and homogeneity of variance, respectively. A multilevel ANOVA followed by Tukey’s post hoc comparisons was used to investigate the effects of different concentrations of CT/HAP NPs, etching modes, and storage times on µTBS outcomes. The tooth/beam clustering was treated as a random effect. Also, a multilevel ANOVA was used to explore the effects of different concentrations of CT/HAP NPs and etching modes on wettability outcomes, followed by Tukey’s post hoc comparisons.

For pH and degree of conversion measurements, one-way ANOVA with Tukey’s post hoc test was employed. Statistical significance was determined at *p* < 0.05 for all tests. All statistical analysis was conducted using R software (version 4.5.0, Windows).

## Results

### Characterization of CT/HAP composite nanoparticles

**Fig. 2:**
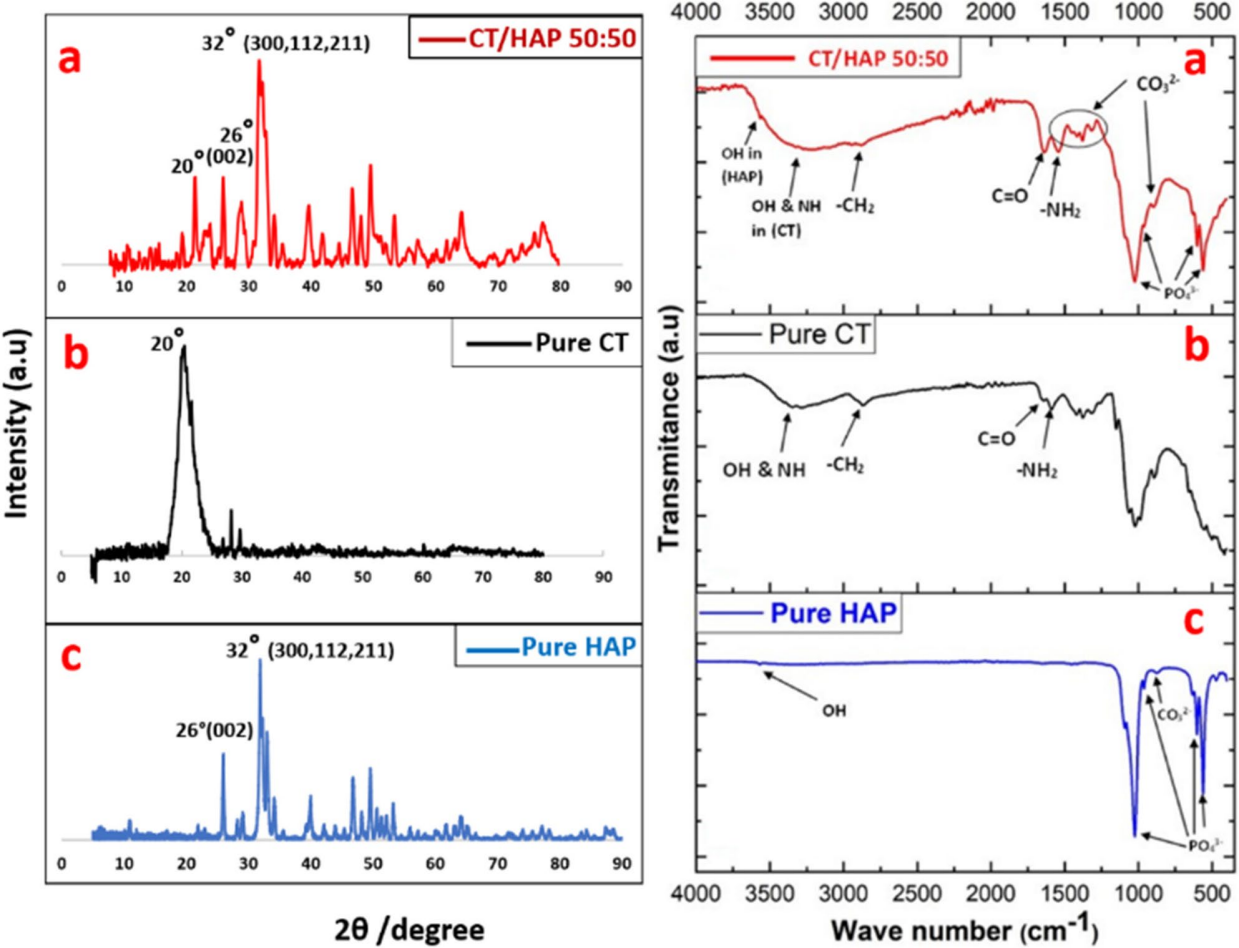
XRD patterns (left) and FTIR spectra (right) of (**a**) CT/HAP NPs (50:50 wt.%), (**b**) pure chitosan (CT), and (**c**) pure nano-hydroxyapatite (nHAP)

X-ray diffraction patterns of nHAP, pure CT, and CT/HAP 50:50 wt% are displayed in (Fig. 2). XRD analysis of the CT/HAP NPs reveals the presence of the nHAP structure, evidenced by a diffraction peak around 26°, corresponding to the (002) plane, and a broad peak observed near 32°, which is attributed to overlapping reflections from the (300), (211), and (112) planes of nHAP. The presence of chitosan in CT/HAP NPs is indicated by a distinct peak approximately at 20°, likely due to the alignment of its polymer chains through intermolecular interactions.

FTIR spectra of nHAP, pure CT, and CT/HAP 50:50 wt% are displayed in (Fig. 2). The FTIR analysis of CT/HAP NPs confirms the presence of the nHAP structure, indicated by absorption bands at 1031, 963, 603, and 565 cm^− 1^, which correspond to different bending and stretching modes of the phosphate group (PO_4_^3−^). Additionally, the absorption band at 3570 cm^− 1^ corresponds to a hydroxyl group (OH) in nHAP [34]. Furthermore, the presence of the chitosan structure in CT/HAP was verified by absorption bands around 2900, 1665, and 1595 cm^− 1^, corresponding to methylene (− CH_2_), amide I (C = O), and amino (− NH_2_) groups, respectively. In addition, a broad band at 3425 cm^− 1^ represents the stretching vibrations of the (-NH) and (-OH) groups in CT [34]. The band observed at approximately 875 cm^− 1^ in both pure nano-hydroxyapatite and CT/HAP nanoparticles was attributed to the carbonate group (CO_3_^2−^), whereas the vibrations between 1417 and 1460 cm^− 1^, also associated with the carbonate group, were more prominent in the CT/HAP NPs, suggesting increased carbonate incorporation [20]. 

### µTBS results

Statistical analysis of the effect of CT/HAP concentration on µTBS showed that group III (1 wt% CT/HAP) exhibited significantly higher µTBS values after 24 h than groups I (control) and II (0.5 wt% CT/HAP), regardless of etching mode. No statistically significant difference was detected between Group I and Group II. After 6 months of storage in SBF, there was a statistically significant difference between all tested groups, with group III showing the highest µTBS, followed by group II, and then group I, which showed the lowest mean value in both ER and SE modes (Table 2). 

**Table 2:** Mean ± standard deviation (SD) values of micro-tensile bond strength (MPa) for different tested groups and time intervals

Time	Etching modes	Group I (Control)	Group II (0.5 wt% CT/HAP)	Group III (1 wt% CT/HAP)
24 h	ER	31.98 ± 4.00^Ba^	32.75 ± 4.29^Bb^	37.50 ± 6.90^Ab^
6 months	ER	26.70 ± 4.26^Cb^	37.89 ± 6.76^Ba^	41.81 ± 7.64^Aa^
24 h	SE	27.29 ± 4.09^Ba^	27.72 ± 3.63^Bb^	32.52 ± 6.61^Ab^
6 months	SE	26.26 ± 4.62^Ca^	38.35 ± 7.41^Ba^	42.10 ± 7.23^Aa^

Values with different uppercase superscripts within the same row are significantly different; values with different lowercase superscripts within the same column for every etching mode are significantly different. A significant level was set at (*p* < 0.05)

Regarding the effect of storage on µTBS, group I showed a significant reduction in µTBS after 6 months in ER mode (*p* = 0.005), with no significant change in SE mode (*p* = 0.577). In contrast, both group II and group III demonstrated significantly higher µTBS mean values after 6 months of storage compared to 24 h in both etching modes (Table 2).

**Fig. 3:**
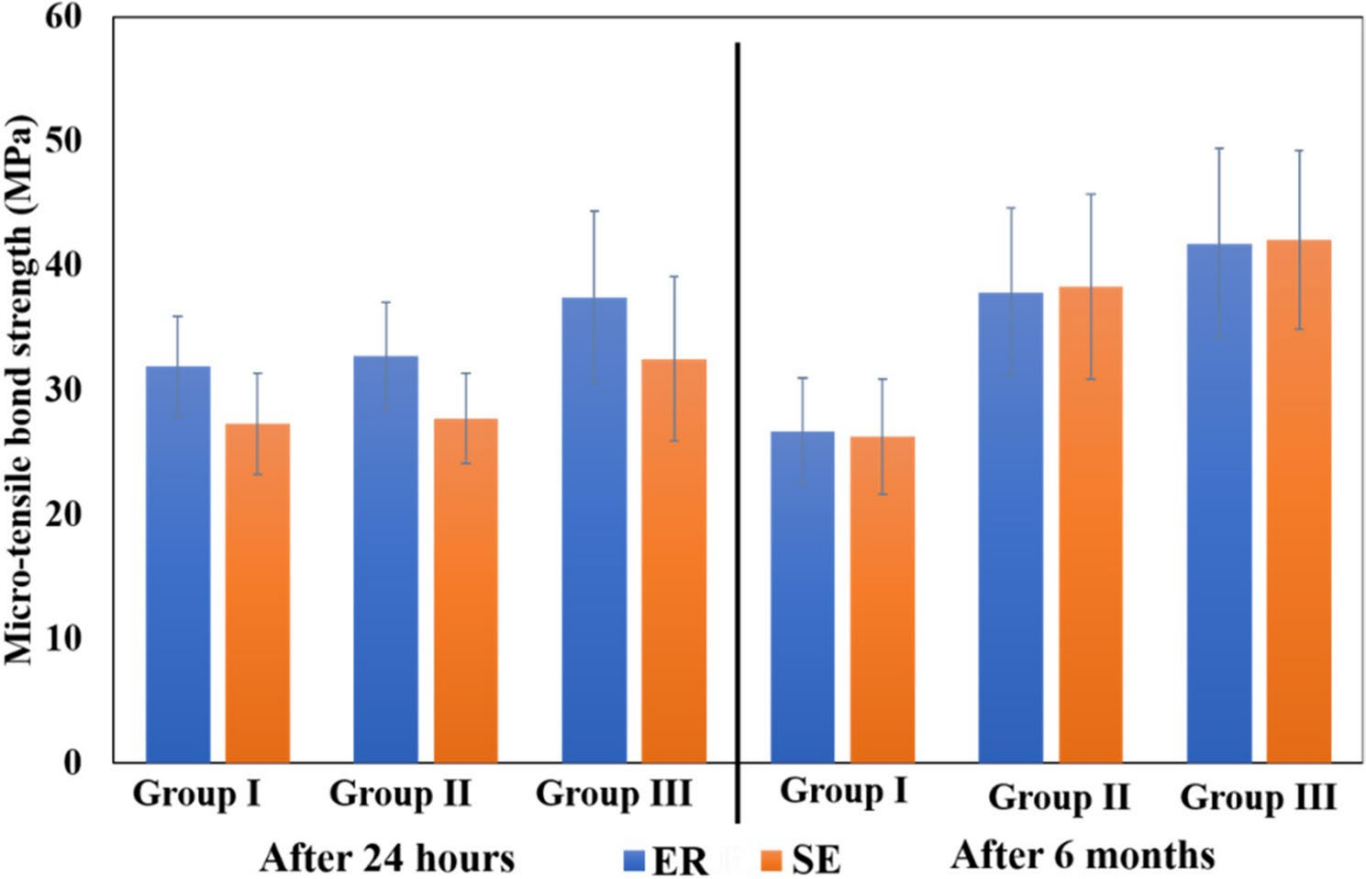
Bar chart showing the mean ± standard deviation (SD) of micro-tensile bond strength for the different groups

Regarding the effect of etching mode, ER produced significantly higher µTBS than SE in all tested groups after 24 h. However, after 6 months, no significant difference was found between the two etching modes (Fig. 3).

**Fig. 4:**
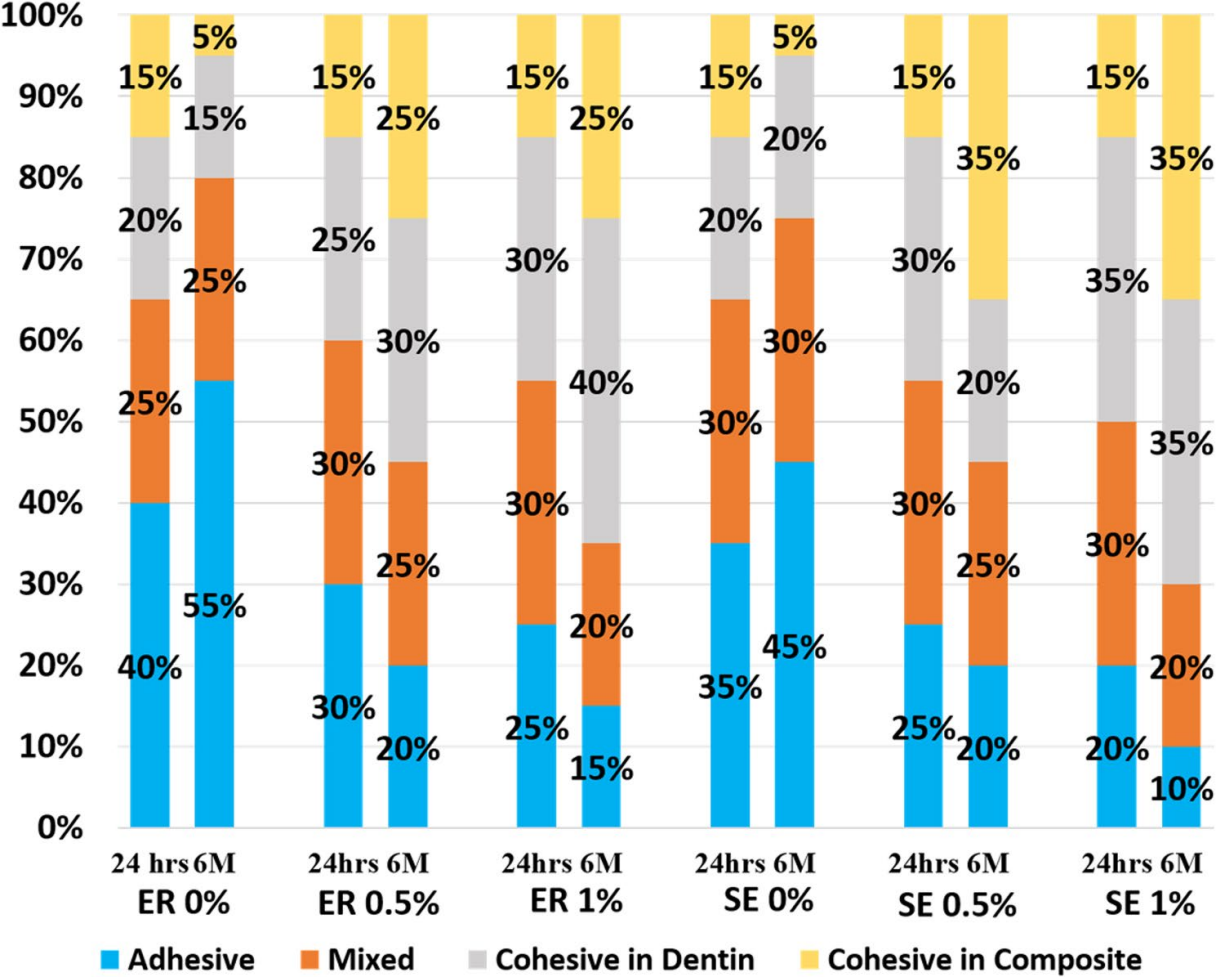
Stacked bar chart illustrating the percentage distribution of failure modes following µTBS testing for all tested groups after 24 h and 6 months of storage

Three-way ANOVA for µTBS demonstrated significant main effects of concentration of CT/HAP composite nanoparticles, etching mode, and storage time. A significant interaction was observed between concentration and storage time (*p* < 0.001). However, the three-way interaction among the tested variables was not significant (Table 3). Failure mode distribution of the deboned specimens for each tested group is shown in Fig. 4. 

**Table 3:** Three-way ANOVA for the effect of different variables and their interactions on micro-tensile bond strength (MPa)

Source	df	Mean Square	f-value	*p*-value
**Concentration of CT/HAP NPs**	2	178.69	5.28	0.006*
**Etching mode**	1	219.92	6.49	0.011*
**Storage time**	1	278.47	8.22	0.005*
Concentration of CT/HAP NPs * Etching mode	2	0.34	0.01	0.990ns
Concentration of CT/HAP NPs * Storage time	2	335.35	9.90	< 0.001*
Etching mode * Storage time	1	90.31	2.67	0.104ns
Concentration of CT/HAP NPs * Etching mode* Storage time	2	2.17	0.06	0.938ns

*df* degree of freedom, * significant (*p* < 0.05);* ns* not significant

### Hybrid layer evaluation

**Fig. 5:**
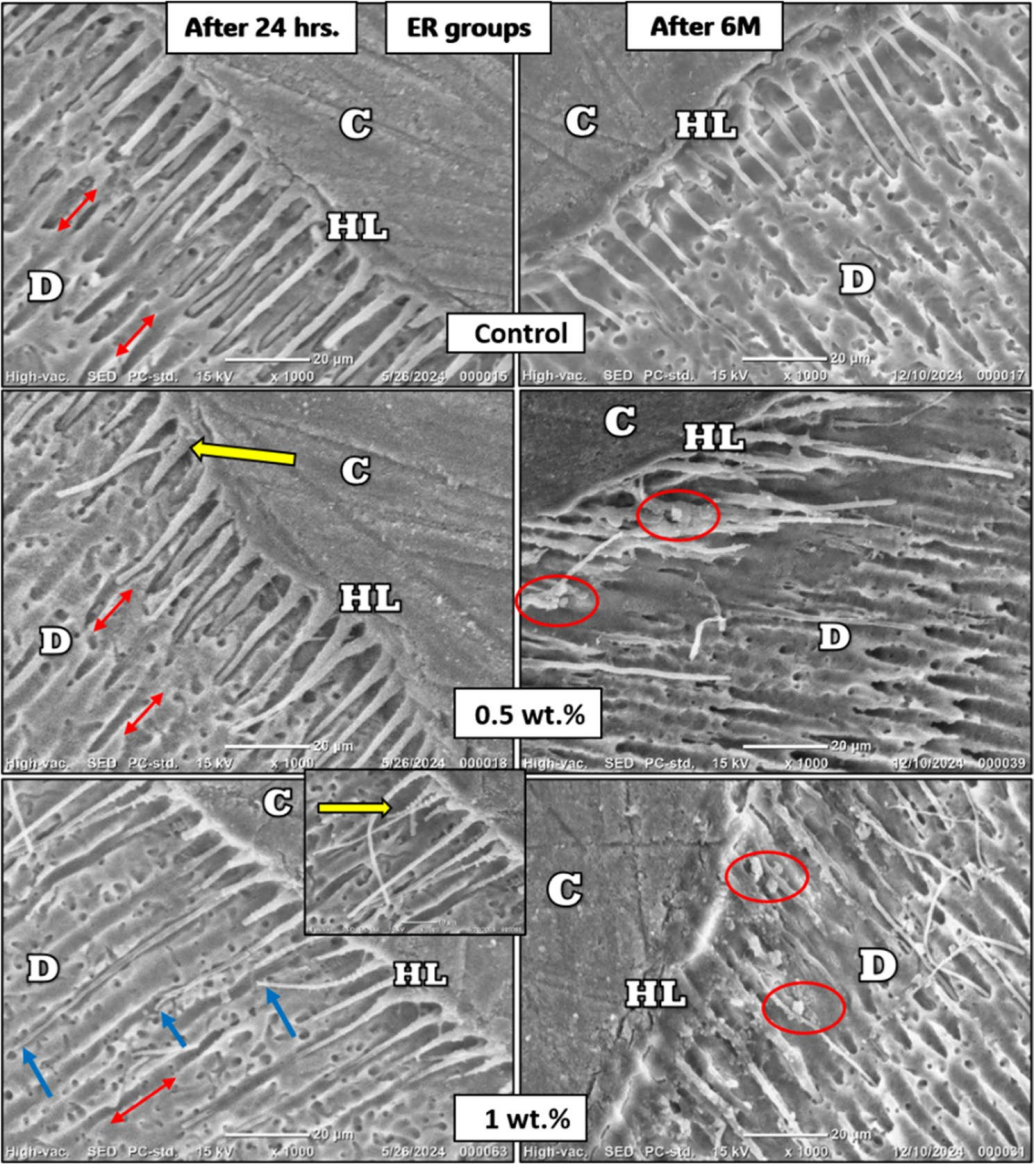
SEM images(1000x) for the hybrid layer of ER groups. C: composite layer; D: dentin layer; HL: hybrid layer. Yellow arrows indicate nanoparticle’s penetration along with the adhesive inside the dentinal tubules, which was clearly evident in the 2000x image of the 1 wt.% group. Red arrows point to interfacial gaps, blue lines indicate inhomogeneity in length of resin tags, while red circles refer to crystal growth after 6 months of storage in SBF

**Fig. 6:**
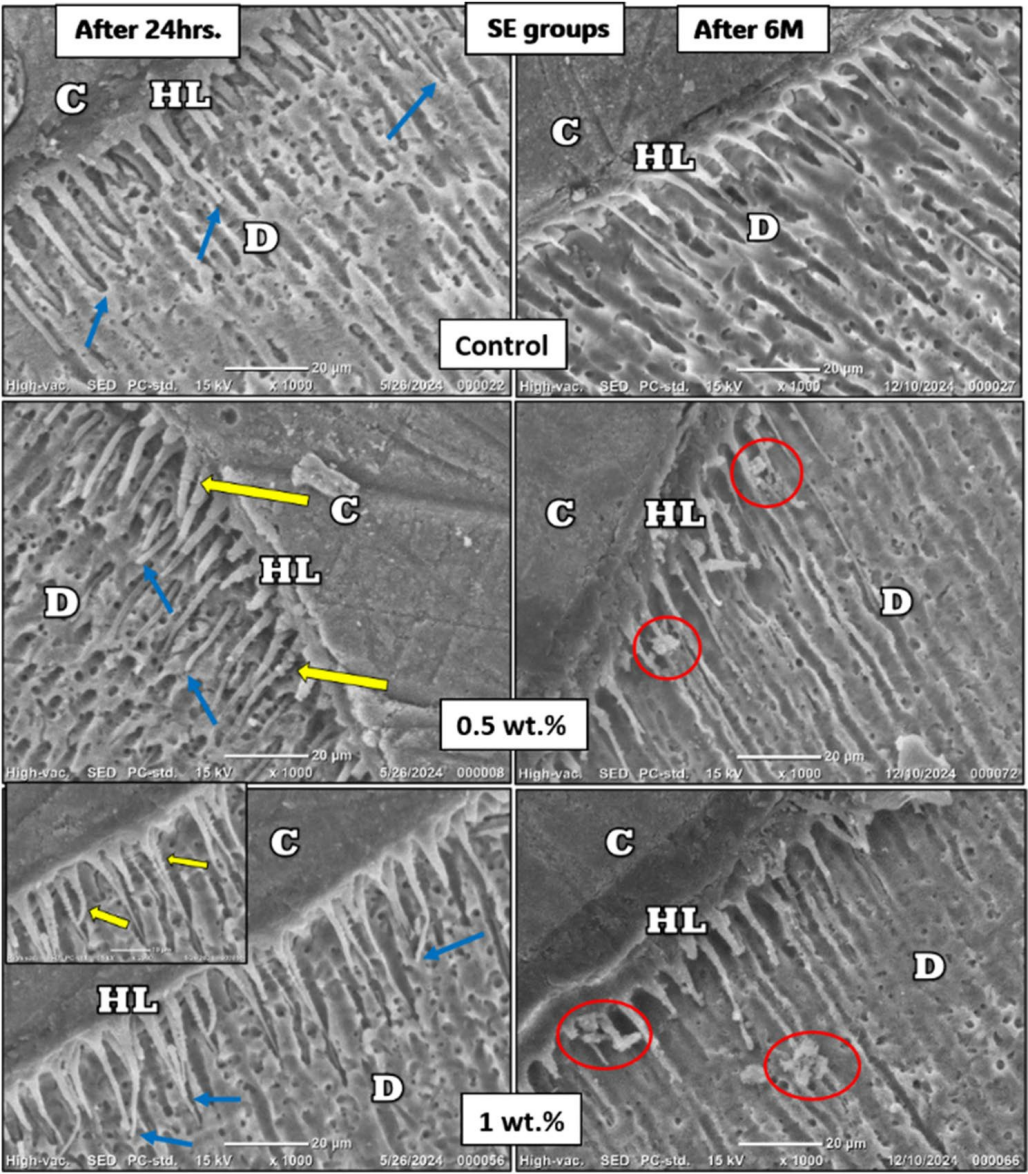
SEM images(1000x) of the hybrid layer in SE groups. C: composite layer; D: dentin layer; HL: hybrid layer. Yellow arrows indicate the nanoparticle’s penetration along with the adhesive inside the dentinal tubules, which was clearly evident in the 2000x image of the 1 wt.% group. Blue lines refer to homogeneity in length of resin tags, and red circles refer to crystal growth after 6 months of storage in SBF

After 24 hours, SEM evaluation of the hybrid layer showed that the ER groups exhibited irregular, inconsistent resin tags and noticeable interfacial gaps, whereas the SE groups demonstrated shorter, more uniform tags with no evident gaps. After 6 months of storage, group I showed resin tag degradation, which was more pronounced in the ER mode, while both group II and III (modified groups) in either ER or SE modes showed crystal-like mineral deposits along the resin tags (Figure 5) and (Figure 6)

### In vitro bioactivity assessment

**Fig. 7:**
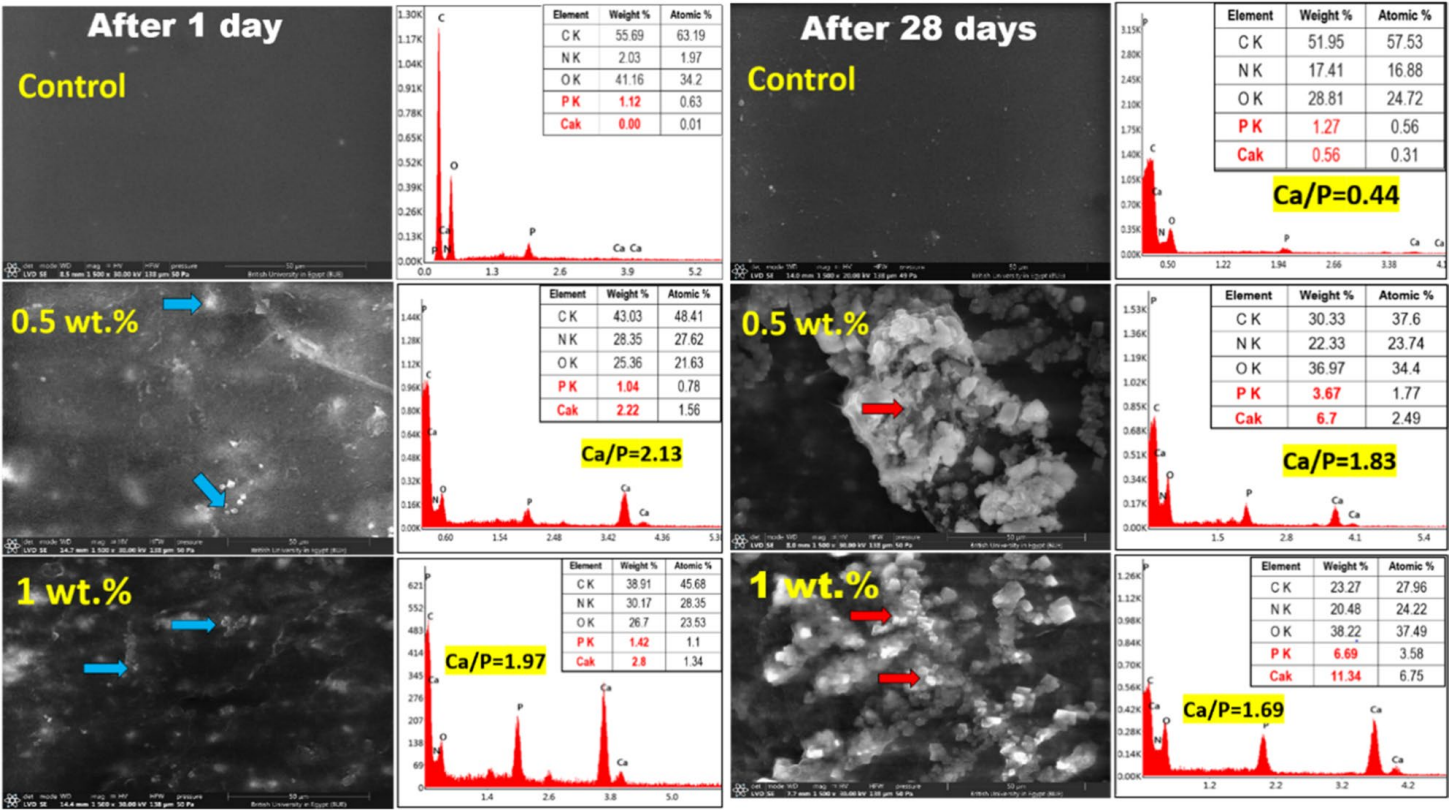
ESEM images of adhesive surfaces at 1500x magnification, along with the corresponding EDX analysis of different tested groups. Blue arrows indicate dispersed white, opaque CT/HAP NPs within dark backgrounds representing the organic matrix. After 28 days, red arrows indicate crystal deposition on the specimen surfaces of modified groups, whereas there was no observable change for the control group

ESEM examination of the adhesive discs after 1 day immersion in SBF revealed a dark background, corresponding to the organic matrix in all groups. In groups II and III (modified groups), opaque white particles representing CT/HAP composite nanoparticles were dispersed within the organic matrix, whereas group I showed no such particles. After 28 days of immersion in SBF, groups II and III showed the formation of crystal aggregates of various sizes on the adhesive discs, with no observable change in group I (Fig. 7).

EDX analysis of modified groups revealed the presence of peaks of calcium and phosphorous, indicating the formation of calcium phosphate precipitates with decreasing calcium/phosphorus ratio over time, approaching a molar ratio of hydroxyapatite (Fig. 7). 

### Wettability, pH, and degree of conversion results

Two-way ANOVA of contact angle measurements revealed that neither the CT/HAP NPs concentration nor the etching mode had a statistically significant effect on the wettability of demineralized dentin, with no significant interaction detected between the tested variables. Accordingly, no statistically significant differences in mean contact angle were observed among the tested groups in both ER (*p* = 0.347) and SE (*p* = 0.114) modes. Similarly, one-way ANOVA of the pH and DC results demonstrated no statistically significant differences among the tested groups (Table 4).

**Table 4:** Mean ± standard deviation (SD) values of contact angle, pH, and degree of conversion for the different tested groups

	Etching modes	Group I (Control)	Group II (0.5 wt.% CT/HAP)	Group III(1 wt.% CT/HAP)
Contact angle (θ)	ER	14.10±1.16	15.43±2.91	15.51±2.44
	SE	14.42±1.68	15.22±1.58	16.69±1.74
pH		3.19±0.17	3.33±0.28	3.50±0.28
DC (%)		77.87±2.62	78.19±4.45	76.76±6.48

For all groups in horizontal and vertical comparisons (*p* *>* 0.05, no significant difference)

## Discussion

The results of this study led to the rejection of the tested null hypotheses. Adding CT/HAP composite nanoparticles enhanced the bonding performance and durability of the adhesive under both ER and SE modes, as well as its bioactivity, while preserving its wettability, pH, and degree of conversion.

Minimally invasive dentistry emphasizes bonding to sound and CAD as part of the peripheral seal concept [25, 35]. While modern adhesives achieve satisfactory immediate bond strength, their long-term durability, particularly to CAD, remains limited [4, 36]. Therefore, improving the durability of resin–dentin bonding to CAD was selected as a key objective of the present study.

To assess the combined benefits of the chitosan polymer and nano-hydroxyapatite, CT/HAP composite nanoparticles were evaluated in this study. A ratio (50:50 wt%) of CT/HAP composite nanoparticles was selected, as this composition reportedly combines the collagen-stability and antimicrobial properties of chitosan with the mineralizing effect of hydroxyapatite [8, 37, 38]. 

CT/HAP composite nanoparticles (50:50 wt%) were prepared through a one-step co-precipitation method using chitosan solution and soluble HAP precursors in aqueous media. Based on previous literature [8, 19], this approach resulted in a more homogeneous dispersion of nHAP within the chitosan matrix compared with traditional blending techniques. This homogeneous distribution promoted stronger interfacial bonding between CT and HAP, which was confirmed by XRD and FTIR characterization.

XRD analysis of CT/HAP NPs revealed slightly broader and less intense peaks compared to those of pure nHAP and chitosan, indicating a slight reduction in crystallinity, as shown in (Fig. 2). This confirmed successful composite formation and structural interaction between CT and HAP [19]. This might be due to the presence of hydrogen bonding and coordination bonds between the hydroxyl groups of HAP and both the hydroxyl and amino groups of chitosan [14, 34]. 

Moreover, FTIR analysis confirmed the presence of characteristic functional groups of both CT and HAP, indicating their successful coexistence and incorporation within the composite structure [19]. The formation of carbonated nHAP was clearly detected after composite nanoparticle synthesis, likely due to the incorporation of carbonate ions during the preparation. This feature is considered advantageous, as carbonated apatite may further enhance the biological activity and bonding capacity of CT/HAP NPs [20]. 

Regarding µTBS results (Table 2), group III (1 wt% CT/HAP) exhibited significantly higher bond strength after 24 h compared to group I (control) and group II (0.5 wt% CT/HAP), irrespective of the etching mode. This improvement is likely due to the strengthening effect of nanoparticles, which serve as fillers and improve the adhesive’s mechanical properties [15, 33]. 

Regarding the effect of time, after 6 months of storage, the control group in ER mode showed a significant decrease in bond strength, whereas SE mode remained relatively stable [36]. In contrast, both modified groups (0.5 wt% and 1 wt% CT/HAP) showed a significant increase in bond strength over time in both etching modes.

The observed enhancement in long-term bond strength could be attributed to the synergistic effect of nHAP-mediated remineralization [12] and CT-induced collagen crosslinking [7], which collectively strengthened the hybrid layer and counteracted aging-related degradation [5, 8, 9]. 

Chitosan plays a crucial role at the resin–dentin interface, contributing to the observed enhancement in µTBS through several mechanical and biochemical mechanisms. Chitosan exhibits selective chelating ability through its amino and hydroxyl functional groups. These functional groups form stable complexes with Ca²⁺ ions present in CAD or released from hydroxyapatite nanoparticles, via coordination chelation (bridge or pendant models) [15]. Additionally, chitosan interacts with dentin collagen through hydrogen bonding, promoting collagen cross-linking and structural stabilization [10]. Calcium chelation may further reduce the availability of metal ions required for MMP activation, thereby limiting collagen degradation over time. These combined mechanisms enhance resin infiltration and preserve hybrid layer integrity, particularly in CAD, ultimately improving long-term bond strength. These findings are consistent with those reported by Baena et al. [39] and Zidan A [40].

Regarding the effect of etching mode (Fig. 3), ER groups exhibited higher µTBS than SE groups for all tested groups after 24 h of storage in SBF. This might be due to deep phosphoric acid etching, which eliminates the smear layer, enhancing dentin permeability, and creating longer resin tags [36]. Nevertheless, after 6 months, no significant difference was recorded between ER and SE modes in all tested groups, suggesting that long-term performance was more influenced by nanoparticle modification than by the etching mode [36, 41]. This finding was consistent with Paschoini et al. [7], who reported that dentin treatment with chitosan enhanced the bond strength and durability of the adhesive interface regardless of the etching mode.

Consistent with the µTBS findings, the failure mode analysis revealed a predominant shift toward cohesive failures in dentin and composite in the modified groups (Fig. 4), indicating enhanced interfacial strength and durable resin–dentin bonding [41]. 

ESEM images and EDX analysis of the modified group surfaces revealed newly formed apatite deposits after immersion for 28 days. This indicates successful mineral deposition and highlights the bioactivity of the CT/HAP nanoparticles. This bioactive behavior could be attributed to the partial dissolution of nHAP particles, followed by the precipitation of an apatite layer on the adhesive surface [42]. 

Previous studies have demonstrated that HAP-based composite nanoparticles exhibited reduced thermodynamic stability and higher dissolution rates than pure HAP nanoparticles, particularly when combined with chitosan [5, 26]. This enhanced solubility is closely related to the lower crystallinity of nHAP, as verified by XRD analysis [8, 12, 42]. The presence of these calcium phosphate crystals suggested the formation of apatite-like precursors, which may promote remineralization and stabilize the adhesive interface in the modified adhesives [31]. Such bioactivity is particularly advantageous in CAD, where mineral replenishment is essential, and may partially explain the improved long-term bond durability observed in the modified groups [23]. 

In addition to enhancing bonding efficacy and bioactivity, it is essential that any modification of dental adhesives preserves their fundamental physicochemical properties. Therefore, the effects of incorporating CT/HAP NPs on the wettability, pH, and degree of conversion of the adhesive were evaluated to ensure that the overall adhesive performance remained uncompromised.

The wettability of demineralized dentin by the tested adhesives was examined in the present study (Table 4). Contact angle measurements revealed no significant difference among the tested groups, which may be attributed to the low viscosity of the selected universal adhesive [15]. In addition to the low concentration of CT/HAP NPs incorporated. This finding aligns with a previous study [43] that reported no significant difference in the wettability of primers modified with 5 wt% or 10 wt% of chitosan-based bioactive fillers.

The pH results obtained in the current study indicated that incorporating CT/HAP NPs into the adhesive did not significantly alter the acidity of the adhesive. This outcome is considered favorable, since the etching potential of the adhesive is largely controlled by its pH [15]. Thus, incorporating a new substance should maintain the acidic etching ability of the universal adhesive, especially in the SE mode. These findings are in agreement with a study performed by Zhang Y and Wang Y [44], who reported that incorporating up to 1 wt% HAP had no significant effect on the pH of the mild self-etch adhesive, owing to its weak acidity and limited ability to undergo neutralization of HAP particles.

The degree of conversion results demonstrated that the addition of 0.5 wt% and 1 wt% CT/HAP NPs did not significantly affect the DC of the adhesive (Table 4). The explanation for this finding could be related to the characteristics of the adhesive used, which contains a low percentage of fillers. The combination of minimal filler loading and the inherently low film thickness of the universal adhesive used (All-Bond Universal) likely minimized interference with light transmission and polymer network formation. Consequently, the incorporation of CT/HAP NPs up to 1 wt% did not significantly affect the adhesive’s viscosity or its polymerization process [15]. 

These findings are further supported by previous studies reporting that the incorporation of nano-hydroxyapatite or chitosan-based fillers at low concentrations (≤ 5 wt%) does not adversely affect the degree of conversion of adhesive resins [45, 46]. 

This study has several limitations that should be considered when interpreting the results. First, the findings are based on an in vitro model, which may not fully replicate the complex conditions of the oral environment. Second, only one type of universal adhesive and limited concentrations of CT/HAP nanoparticles were evaluated, which may restrict the generalizability of the results. In addition, the remineralizing potential of the modified adhesive was not directly assessed on caries-affected dentin.

Future studies should focus on evaluating the remineralization potential of CT/HAP-modified adhesives directly on caries-affected dentin, using different adhesive systems, different ratios and concentrations of CT/HAP nanoparticles, as well as assessing their durability under extended storage periods and simulated oral conditions, including thermomechanical cycling.

## Conclusions

Within the limitations of this in vitro study, the following conclusions could be drawn:

- Incorporation of 0.5 wt% and 1 wt% chitosan/hydroxyapatite composite nanoparticles into an ultra-mild universal adhesive significantly improved bonding stability to caries-affected dentin, while maintaining its wettability, pH, and degree of conversion.
- Incorporation of 0.5 wt% and 1 wt% chitosan/hydroxyapatite composite nanoparticles into an ultra-mild universal adhesive improved the hybrid layer’s resistance to degradation in both etch-and-rinse and selective-etch modes.
- Incorporation of 0.5 wt% and 1 wt% chitosan/hydroxyapatite composite nanoparticles into an ultra-mild universal adhesive enhanced the in vitro bioactivity of the adhesive.

## Data Availability

The datasets used and/or analysed during the current study are available from the corresponding author upon reasonable request.

## Abbreviations

CT/HAP: NPsChitosan/hydroxyapatite composite nanoparticles
CAD: Caries-affected dentin
ER: Etch-and-Rinse mode
SE: Selective-Etch mode
µTBS: Micro-tensile bond strength
SBF: Simulated body fluid
ESEM: Environmental scanning electron microscope
EDX: Energy-dispersive X-ray spectroscopy
DC: Degree of conversion
FTIR: Fourier transform infrared spectroscopy
MPa: Megapascal
CID: Caries-infected dentin
CAD: Caries-affected dentin
MID: Minimally invasive dentistry
MMPs: Matrix Metalloproteinases
CT: Chitosan
HAP: Hydroxyapatite
nHAP: Nano-hydroxyapatite

## References

[1] Nisar S, Hass V, Wang Y. Effects of crosslinker-modified etchants on durability of resin-dentin bonds in sound and caries-affected dentin. Dent Mater. 2025;41(5):575–83. 10.1016/j.dental.2025.03.005. 40118707 10.1016/j.dental.2025.03.005 PMC11994283

[2] Cascales ÁF, Moscardó AP, Toledano M, Banerjee A, Sauro S. An in-vitro investigation of the bond strength of experimental ion-releasing dental adhesives to caries-affected dentine after 1 year of water storage. J Dent. 2022;119:104075. 10.1016/j.jdent.2022.104075. 35227835 10.1016/j.jdent.2022.104075

[3] Lim ZE, Duncan HF, Moorthy A, McReynolds D. Minimally invasive selective caries removal: a clinical guide. Br Dent J. 2023;234(4):233–40. 10.1038/s41415-023-5515-4. 36829011 10.1038/s41415-023-5515-4 PMC9957719

[4] Mohanty PR, Mishra L, Saczuk K, Lapinska B. Optimizing adhesive bonding to caries affected dentin: A comprehensive systematic review and Meta-Analysis of dental adhesive strategies following Chemo-Mechanical caries removal. Appl Sci. 2023;13(12):7295. 10.3390/app13127295.

[5] Saravana Karthikeyan B, Mahalaxmi S. Biomimetic dentin remineralization using eggshell derived nanohydroxyapatite with and without carboxymethyl chitosan — An in vitro study. Int J Biol Macromol. 2024;270:132359. 10.1016/j.ijbiomac.2024.132359. 38754678 10.1016/j.ijbiomac.2024.132359

[6] Al Wadei MHD, Ahmed SZ, Almoallim MH, Towaireet FM, Hamad T, Agwan MA, et al. Universal adhesive fortified with inorganic nanoparticles on dentin affected by caries: A comprehensive study utilizing SEM, EDX, Micro-Tensile bond strength and antimicrobial effectiveness. Microsc Res Tech. 2025;88(6):1848–57. 10.1002/jemt.24816. 39982848 10.1002/jemt.24816

[7] Paschoini VL, Ziotti IR, Neri CR, Corona SAM, Souza-Gabriel AE. Chitosan improves the durability of resin-dentin interface with etch-and-rinse or self-etch adhesive systems. J Appl Oral Sci. 2021;29. 10.1590/1678-7757-2021-0356. 10.1590/1678-7757-2021-0356 PMC8687651 34910075

[8] Bushra A, Subhani A, Islam N. A comprehensive review on biological and environmental applications of chitosan-hydroxyapatite biocomposites. Compos Part C Open Access. 2023;12:100402. 10.1016/j.jcomc.2023.100402.

[9] Li Z, Zeng Y, Ren Q, Ding L, Han S, Hu D, et al. Mineralization promotion and protection effect of carboxymethyl Chitosan biomodification in biomimetic mineralization. Int J Biol Macromo. 2023;234:123720. 10.1016/j.ijbiomac.2023.123720. 10.1016/j.ijbiomac.2023.123720 36805508

[10] Nimbeni SB, Nimbeni BS, Divakar DD. Role of Chitosan in remineralization of enamel and dentin: A systematic review. Int J Clin Pediatr Dent. 2021;14(4):562–8. 10.5005/jp-journals-10005-1971. 34824515 10.5005/jp-journals-10005-1971 PMC8585910

[11] Pushpalatha C, Gayathri VS, Sowmya SV, Augustine D, Alamoudi A, Zidane B, et al. Nanohydroxyapatite in dentistry: A comprehensive review. Saudi Dent J. 2023;35(6):741–52. 10.1016/j.sdentj.2023.05.018. 37817794 10.1016/j.sdentj.2023.05.018 PMC10562112

[12] Hassaan M, Rashad N, El Sawa A, Sedik A. Efficacy of hydroxyapatite nanoparticles in dentinal tubule occlusion and resistance to erosive wear (scanning electron microscopic study). Alex Dent J. 2024;49(1):57–65. 10.21608/adjalexu.2023.211107.1379.

[13] Stafin K, Śliwa P, Pia Tkowski M, Matýsek D. Chitosan as a templating agent of calcium phosphate crystalline phases in biomimetic mineralization: theoretical and experimental studies. ACS Appl Mater Interfaces. 2024;16(46):63155–69. 10.1021/acsami.4c11887. 39526983 10.1021/acsami.4c11887

[14] Soriente A, Fasolino I, Gomez-Sánchez A, Prokhorov E, Buonocore GG, Luna-Barcenas G, et al. Chitosan/hydroxyapatite nanocomposite scaffolds to modulate osteogenic and inflammatory response. J Biomed Mater Res A. 2022;110(2):266–72. 10.1002/jbm.a.37283. 34331513 10.1002/jbm.a.37283 PMC9291049

[15] Ezz El-Din Y, El-Banna A, Hussein TS. Bonding of Chitosan and Nanochitosan modified universal adhesive to dentin. Int J Adhes Adhes. 2023;125. 10.1016/j.ijadhadh.2023.103432.

[16] Daood U, Fawzy A. Development of a bioactive dentin adhesive resin modified with magnesium-doped synthetic hydroxyapatite crystals. J Mech Behav Biomed Mater. 2023;140. 10.1016/j.jmbbm.2023.105737. 10.1016/j.jmbbm.2023.105737 36827934

[17] Elasser DM, Niazy MA, Elsharkawy DA, Mansour MS. The remineralizing potential of nano bioactive glass versus nanohydroxyapatite on dentine as affected by pH cycling. Al-Azhar Dent J Girls. 2018;5(4):327–34.

[18] Mohammed N, Motawea I, Eltayeb H. Effect of incorporation of hydroxyapatite nanorods on the rheological properties, micro-shear bond strength and degree of conversion of two dental adhesives. Al-Azhar Dent J Girls. 2017;4(3):205–13.

[19] Nikpour MR, Rabiee SM, Jahanshahi M. Synthesis and characterization of hydroxyapatite/chitosan nanocomposite materials for medical engineering applications. Compos B Eng. 2012;43(4):1881–6. 10.1016/j.compositesb.2012.01.056.

[20] Andrade Neto DM, Carvalho EV, Rodrigues EA, Feitosa VP, Sauro S, Mele G, et al. Novel hydroxyapatite nanorods improve anti-caries efficacy of enamel infiltrants. Dent Mater. 2016;32(6):784–93. 10.1016/j.dental.2016.03.026. 27068739 10.1016/j.dental.2016.03.026

[21] Devine DM, Hoctor E, Hayes JS, Sheehan E, Evans CH. Extended release of proteins following encapsulation in hydroxyapatite/chitosan composite scaffolds for bone tissue engineering applications. Mater Sci Eng C Mater Biol Appl. 2018;84:281–9. 10.1016/j.msec.2017.11.001. 29519440 10.1016/j.msec.2017.11.001 PMC5846124

[22] Becerra J, Rodriguez M, Leal D, Noris-Suarez K, Gonzalez G. Chitosan-collagen-hydroxyapatite membranes for tissue engineering. J Mater Sci Mater Med. 2022;33(2):18. 10.1007/s10856-022-06643-w. 35072812 10.1007/s10856-022-06643-w PMC8786760

[23] Kazem NE, El-Refai DA, Alian G. Assessment of physical properties of bioactive glass-modified universal multimode adhesive and its bonding potential to artificially induced caries affected dentin. BMC Oral Health. 2024;24(1). 10.1186/s12903-024-04175-z. 10.1186/s12903-024-04175-z PMC10998361 38580948

[24] Elsaka SE. Antibacterial activity and adhesive properties of a chitosan-containing dental adhesive. Quintessence Int. 2012;43(7):603–13. 22670256

[25] Elmalawany LM, Sherief DI, Alian GA. Theobromine versus casein phospho-peptides/Amorphous calcium phosphate with fluoride as remineralizing agents: effect on resin-dentine bond strength, microhardness, and morphology of dentine. BMC Oral Health. 2023;23(1):447. 10.1186/s12903-023-03139-z. 37403039 10.1186/s12903-023-03139-z PMC10318693

[26] Chen Z, Cao S, Wang H, Li Y, Kishen A, Deng X, et al. Biomimetic remineralization of demineralized dentine using scaffold of CMC/ACP nanocomplexes in an in vitro tooth model of deep caries. PLoS ONE. 2015;10(1):e0116553. 10.1371/journal.pone.0116553. 25587986 10.1371/journal.pone.0116553 PMC4294661

[27] Smith PW, Preston KP, Higham SM. Development of an in situ root caries model. A. In vitro investigations. J Dent. 2005;33(3):253–67. 10.1016/j.jdent.2004.10.020. 15725525 10.1016/j.jdent.2004.10.020

[28] Moloney E, Varanasis S, Meyers l, Rintoul L, Symons A. The effect of remineralisation treatments on demineralised dentine, an in vitro study. Open J Dent Oral Med. 2014;2(1):1–8. 10.13189/ojdom.2014.020101.

[29] Armstrong S, Breschi L, Özcan M, Pfefferkorn F, Ferrari M, Van Meerbeek B. Academy of dental materials guidance on in vitro testing of dental composite bonding effectiveness to dentin/enamel using micro-tensile bond strength (µTBS) approach. Dent Mater. 2017;33(2):133–43. 10.1016/j.dental.2016.11.015. 28007396 10.1016/j.dental.2016.11.015

[30] Sasikumar Y, Kumar AM, Babu RS, Rahman MM, Samyn LM, de Barros ALF. Biocompatible hydrophilic brushite coatings on AZX310 and AM50 alloys for orthopaedic implants. J Mater Sci Mater Med. 2018;29(8):123. 10.1007/s10856-018-6131-8. 30032462 10.1007/s10856-018-6131-8

[31] Carneiro KK, Araujo TP, Carvalho EM, Meier MM, Tanaka A, Carvalho CN, et al. Bioactivity and properties of an adhesive system functionalized with an experimental niobium-based glass. J Mech Behav Biomed Mater. 2018;78:188–95. 10.1016/j.jmbbm.2017.11.016. 29169095 10.1016/j.jmbbm.2017.11.016

[32] Elkassaby A, Kandil M, Alian G. Microtensile vs. Flexural bond strength for bond strength assessment. J Res Med Dent Sci. 2022;10:53–8.

[33] Alhenaki AM, Attar EA, Alshahrani A, Farooq I, Vohra F, Abduljabbar T. Dentin bond integrity of filled and unfilled resin adhesive enhanced with silica Nanoparticles—An SEM, EDX, Micro-Raman, FTIR and Micro-Tensile bond strength study. Polymers. 2021;13(7):1093. 10.3390/polym13071093. 33808159 10.3390/polym13071093 PMC8037508

[34] Ying R, Wang H, Sun R, Chen K. Preparation and properties of a highly dispersed nano-hydroxyapatite colloid used as a reinforcing filler for Chitosan. Mater Sci Eng C Mater Biol Appl. 2020;110:110689. 10.1016/j.msec.2020.110689. 32204004 10.1016/j.msec.2020.110689

[35] Isolan CP, Sarkis-Onofre R, Moraes RR. Bonding to sound and caries-affected dentin: systematic review and meta-analysis. Dent Mater. 2015;31:e55. 10.1016/j.dental.2015.08.122. 29399679 10.3290/j.jad.a39775

[36] Cuevas-Suárez C, da Rosa W, Lund R, da Silva A, Piva E. Bonding performance of universal adhesives: an updated systematic review and Meta-Analysis. J Adhes Dent. 2019;21(1):7–26. 10.3290/j.jad.a41975. 30799468 10.3290/j.jad.a41975

[37] Rogina A, Rico P, Gallego Ferrer G, Ivanković M, Ivanković H. In situ hydroxyapatite content affects the cell differentiation on porous Chitosan/Hydroxyapatite scaffolds. Ann Biomed Eng. 2016;44(4):1107–19. 10.1007/s10439-015-1418-0. 26265459 10.1007/s10439-015-1418-0

[38] Gurucharan I, Isaac DRD, Madhubala MM, Amirtharaj LV, Mahalaxmi S, Jayasree R, et al. Effect of Chitosan and hydroxyapatite nanocomposite on dentin erosion: an in-vitro study. J Int Oral Health. 2022;14(5):509–17. 10.4103/jioh.jioh_50_22.

[39] Baena E, Cunha SR, Maravić T, Comba A, Paganelli F, Alessandri-Bonetti G, et al. Effect of Chitosan as a Cross-Linker on matrix metalloproteinase activity and bond stability with different adhesive systems. Mar Drugs. 2020;18(5):263. 10.3390/md18050263. 32443628 10.3390/md18050263 PMC7280998

[40] Zidan A. Effect of Chitosan on resin-dentin interface durability: A 2 year in-vitro study. Egypt Dent J. 2019;65(3):2955–65. 10.21608/edj.2019.72691.

[41] Elkaffas AA, Hamama HH, Mahmoud SH, Fawzy AS. Effect of acid etching on dentin bond strength of ultra-mild self-etch adhesives. Int J Adhes Adhes. 2020;99:102567. 10.1016/j.ijadhadh.2020.102567.

[42] Mohandes F, Salavati-Niasari M. Freeze-drying synthesis, characterization and in vitro bioactivity of chitosan/graphene oxide/hydroxyapatite nanocomposite. RSC Adv. 2014;4(49):25993–6001. 10.1039/c4ra03534h.

[43] Riyadh Al-Banaa L, Al-Khatib AR, Habeeb Jabrail F. Evaluation of the biological, physical, mechanical and chemical properties of orthodontic primer modified by nano-chitosan loaded with bioactive materials. J Oral Biol Craniofac Res. 2025;15(3):500–7. 10.1016/j.jobcr.2025.03.002. 40160849 10.1016/j.jobcr.2025.03.002 PMC11953976

[44] Zhang Y, Wang Y. Hydroxyapatite effect on photopolymerization of self-etching adhesives with different aggressiveness. J Dent. 2012;40(7):564–70. 10.1016/j.jdent.2012.03.005. 22445789 10.1016/j.jdent.2012.03.005 PMC3367082

[45] Leitune VCB, Collares FM, Trommer RM, Andrioli DG, Bergmann CP, Samuel SMW. The addition of nanostructured hydroxyapatite to an experimental adhesive resin. J Dent. 2013;41(4):321–7. 10.1016/j.jdent.2013.01.001. 23313828 10.1016/j.jdent.2013.01.001

[46] Machado AHS, Garcia IM, Motta AdSd, Leitune VCB, Collares FM. Triclosan-loaded Chitosan as antibacterial agent for adhesive resin. J Dent. 2019;83:33–9. 10.1016/j.jdent.2019.02.002. 30794843 10.1016/j.jdent.2019.02.002

